# Extant thrips diverged in the early tertiary period

**DOI:** 10.1186/s12863-023-01146-1

**Published:** 2023-08-16

**Authors:** Chunying Wu, Hongrui Zhang

**Affiliations:** 1 Plant Protection College, Yunnan Agricultural University, Kunming, 650201 P.R. China; 2 State Key Laboratory for Conservation and Utilization of Bio-Resources in Yunnan, Yunnan Agricultural University, Kunming, 650201 China; 3 State Key Laboratory of Genetic Resources and Evolution, Kunming Institute of Zoology, Chinese Academy of Sciences, Kunming, 650201 China

**Keywords:** Thysanoptera, Thrips, Protein-coding gene, Phylogeny, Divergence time

## Abstract

Thysanoptera, commonly known as thrips, are diverse tiny insects whose earliest fossil record dates back to the Triassic period. Since there are few studies on the divergence time of taxa under Thysanoptera, this study used 13 mitochondrial coding protein genes to reconstruct the phylogenetic tree with divergence time of 26 species of this order and show a reliable phylogenetic relationship of thrips species. The time tree of this study shows that most extant thrips diverged in the early Tertiary period, while the fossil records also support that most extinct thrips appeared in this period. This study expands our understanding of the evolution of thrips and provides a feasible way of using multiple mitochondrial genes to establish robust phylogenetic relationships and explore divergence time between species.

**Supplementary Information:**

The online version contains supplementary material available at 10.1186/s12863-023-01146-1.

## Introduction

The insects of the order Thysanoptera Haliday, 1836 are commonly known as thrips. They are widely distributed all over the world, and has a complex diet, mainly including phytophagia, mycophagia and predacia, among which phytophagia accounts for more than half. Thrips are one of the important economic pests. According to its morphological characteristics, this order are divided into two suborders: Terebrantia Haliday, 1836 and Tubulifera Haliday, 1836. Thysanoptera are divided into nine families and currently have more than 6,400 extant species and 187 fossil species (thrips wiki, 2023). Phylogenetic analysis facilitates the identification of taxonomic category divergence during the evolutionary process and interrelationships among them, thereby providing essential information for studying the phylogenetic evolutionary history of insects and explaining the intricate relationships in the evolutionary history of species.

The thrips fossil record shows that this kind of insect appeared very early. *Triassothrips virginicus* [1, 2] found in Virginia-North Carolina border, USA, was recorded from the end of Triassic. *Liassothrips crassipes* Martynov, 1927 and *Karataothrips jurassicus* Sharov, 1972 both found in Kazakhstan, were recorded from the Late Jurassic [3]. A small number of thrips fossils have been found in the Cretaceous. Many of the fossils of extinct thrips species found so far exist at 38 − 13 Mya (https://paleobiodb.org). The specimen *Triassothrips virginicus* was used as a calibration (237 − 207 Mya) for Thysanoptera by Misof [4]. His study speculated that the divergence times of the genus *Gynaikothrips* in family Phlaeothripidae and the genus *Franklinothrips* in family Aeolothripidae was 119.93 Mya, and the genus *Franklinothrips* and the genus *Thrips* in family Thripidae was 54.76 Mya. Using the stem age of Thysanoptera (260 Mya), Pakrashi et al. [5] inferred that the origin time for Thysanoptera was at around 234 Mya, and the suborders Terebrantia and Tubulifera diversify at aound 169 Mya. Yan-hui Wang [6] estimated the insect divergence time using 5 nuclear genes and 13 mitochondrial protein-coding genes from 42 representatives of all 30 insect orders. *Tethysthrips libanicus* was used as the fossil calibration 129.4–125.0 Mya for Thysanoptera, and the two suborders Tubulifera and Terebrantia of Thysanoptera diverged at 127.2 Mya.

Misof [4] selected 144 taxa representing from all extant insect orders and other arthropods for a phylogenomic study, and the results showed that insects originated about 479 Mya [4], almost at the same time as land plants. Insects and land plants jointly shaped the earliest land ecosystems. Their study also shows that the ability to fly in insects originated about 406 Mya [4], long before other animals and around the same time that land plants began to colonize forests on a large scale. The divergence time inside many insect taxa are now well studied. However, the divergence time inside Thysanoptera is not clear. In this background, it will be very interesting to further explore the relationship between species divergence in Thysanoptera in detail. It can bring a new perspective to observe the historical evolution of thrips. But all the currently available fossil records of thrips are extinct species, so we use the earliest fossils of a taxa to label the crown nodes that contain the major branches of this taxa. On the basis of previous studies, we hope to further infer the differentiation time of different families, genera and species in the order Thysanoptera. The earlist fossil calibrations of *Rohrthrips libanicus* (130.0-125.45 Mya) of the family Phlaeothripidae and *Fusithrips crassipes* (125.0-113.0 Mya) of the family Aeolothripidae were used and combined with phylogenetic relationships based on 13 mitochondrial protein-coding genes to infer the divergence time from 26 species in 4 families of Thysanoptera.

## Materials and methods

### Data source and processing

The published mitochondrial genome data of the whole Thysanoptera were searched and downloaded from the Nucleotide database at NCBI (National Center for Biotechnology Information, USA) in November 2022. By manually cleaning the genome data of duplicate or redundant species, the available mitochondrial genome data of 26 species was finally obtained. In PhyloSuite [7] programme, the codon table of invertebrate mitochondrial was chosen and the 13 protein-coding genes (PCGs) were extracted from the mitochondrial genome sequences. The genes were *ATP6, ATP8, COI, COII, COIII, Cytb, ND1, ND2, ND3, ND4L, ND4, ND5 ,ND6*. Homology of sequences was the basis of phylogenetic analysis, so these 13 PCG sequences were aligned in batches with MAFFT [8] using codon alignment mode. Since MACSE used the classic “Needleman-Wunsch” algorithm to correctly identify pseudogenization events and maintain the ancestral codon structure, this study also used MACSE to optimize the PCGs sequence after the alignment of MAFFT. The alignments were refined using the codon optimization program MACSE v. 2.03 [9], which preserves reading frame and allows incorporation of sequencing errors or sequences with frameshifts.

The accuracy of sequence alignment is very important for downstream phylogenetic analysis. Pruning sequences can remove sites with alignment errors or multiple substitutions to remove phylogenetic noise and retain phylogenetic signals, thus improving the accuracy and speed of downstream analysis. The “Codon” mode of Gblock can be sequenced in triplet codon form, therefore the sequences were optimized with Gblocks [10] to obtain the alignment sequences of the optimized conserved sites. Ambiguously aligned fragments of 13 PCGs were removed in batches using Gblocks with the following parameter settings: minimum number of sequences for a conserved/flank position (45/45), maximum number of contiguous non-conserved positions (8), minimum length of a block (10), allowed gap positions (with half). The conserved/flank position (45/45) parameter setting in Gblocks specifies that a position must be present in at least 45 sequences to be considered conserved and that the position must be flanked by at least 45 conserved positions on both sides. The maximum number of contiguous non-conserved positions (8) parameter setting in Gblocks specifies that contiguous non-conserved positions greater than 8 will be rejected. The minimum length of a block (10) parameter setting specifies that blocks smaller than 10 after gap cleaning will be rejected. After optimizing multi-gene alignments, the “concatenate sequence” function in PhyloSuite was used to concatenate the 13 PCG sequences from 26 species.

### Phylogenetic reconstruction

#### ML tree

The maximum likelihood (ML) method is one of the most widely used methods for phylogenetic tree reconstruction. It is based on the principle of finding the tree that maximizes the likelihood of the observed data given a model of evolution. The ML method is preferred over other methods because it is more accurate and efficient in estimating phylogenetic trees. The multi-gene maximum likelihood phylogenetic tree was reconstructed using IQ-TREE [11–13] which is a widely used and open-source software package for phylogenetic inference using the maximum likelihood criterion. The optimal partition strategy and the optimal substitution model were completed by its own ModelFinder [14]. The software automatically selected the optimal partition model and reconstructed the tree, generating 20,000 samples for ultrafast bootstrap.

#### BI tree

MrBayes is a widely used software package for Bayesian inference (BI) of phylogenetic trees. It uses Markov Chain Monte Carlo (MCMC) methods to estimate the posterior probability distribution of trees given the data and a model of evolution. The MCMC method is based on the principle of sampling trees from the posterior probability distribution. MrBayes is preferred over other methods because it can handle complex models of evolution and can estimate the uncertainty in the phylogenetic tree. MrBayes 3.2.6 [15] was used to infer phylogenies using the partition model (2 parallel runs; 2,000,000 generations), with the first 25% of sampled data discarded as burn-in. The optimized multi-gene concatenate sequences were used for gene-based dataset partitioning [16, 17] and optimal model selection. For the parameters, branch lengths were “linked”, models were “mrbayes”, model selection was AICc, and search was greedy. AICc stands for Akaike Information Criterion corrected for small sample sizes. It is a statistical method used for model selection in the context of phylogenetic analysis. Greedy algorithms are a class of algorithms that make locally optimal choices at each step with the hope of finding a global optimum. The results of dataset partitioning and optimal model selection were imported into MrBayes, and the multi-gene phylogenetic tree was reconstructed by Bayesian method. Average standard deviation of split frequencies below 0.01 was used to test whether the BI tree converged.

### Divergence time estimation

The optimized partition sequences were parametrically configured with BEAUTi included in the Bayesian Evolutionary Analysis by Sampling Trees (BEAST, v1.10.4; [18]) package to generate the BEAST software file (.XML format) without setting a starting tree. Divergence time estimation uses the widely recognized timing method based on the Relaxed Clock Log Normal model [19], and the selected model was determined in ModelFinder [14] according to Bayesian Information Criterion (BIC). *Rohrthrips libanicus* (130.0-125.45 Mya) of the family Phlaeothripidae and *Fusithrips crassipes* (125.0-113.0 Mya) of the family Aeolothripidae were used as the earlist fossil calibrations. In order to obtain reliable results in the BEAST analysis, MCMC (Markov chain Monte Carlo) was set to run Chain Length = 100,000,000 generations, sampling once every 10,000 generations and discarding the first 25% aged samples in each subset. The generated BEAST file was submitted to CIPRES (https://www.phylo.org/)[20] online platform for a dated phylogenetic tree construction by the toolkit of BEAST (current) on XSEDE (1.10.4/1.10.5pre). The constructed datad phylogenetic tree used the TreeAnnotator v1.8.4 [21] in the package to generate a maximum clade credibility tree with the information of divergence time and 95% highest posterior density (HPD) confidence interval. Then Tracer v1.7.2 [22] was used to check the effective sample size (ESS), and the convergence of each run was evaluated by confirming that the ESS was greater than 200.

## Results

### DNA sequence data

The total length of the optimized and concatenated sequences is 10,503 bp. *ATP6* 1-657 bp, *ATP8* 658-804 bp, *COI* 805-2322 bp, *COII* 2323-2955 bp, *COIII* 2956-3720 bp, *Cytb* 3721-4821 bp, *ND1* 4822-5733 bp, *ND2* 5734-6594 bp, *ND3* 6595-6942 bp, *ND4L* 6943-7203 bp, *ND4* 7204-8463 bp, *ND5* 8464-10074 bp, *ND6* 10075-10503 bp. There were 2362 conserved sites (22.49%), 8141 variable sites (77.51%), and 7418 parsimony informative sites (70.63%) in one sequence.

### Phylogenetic analysis

#### ML and BI trees

**Fig. 1:**
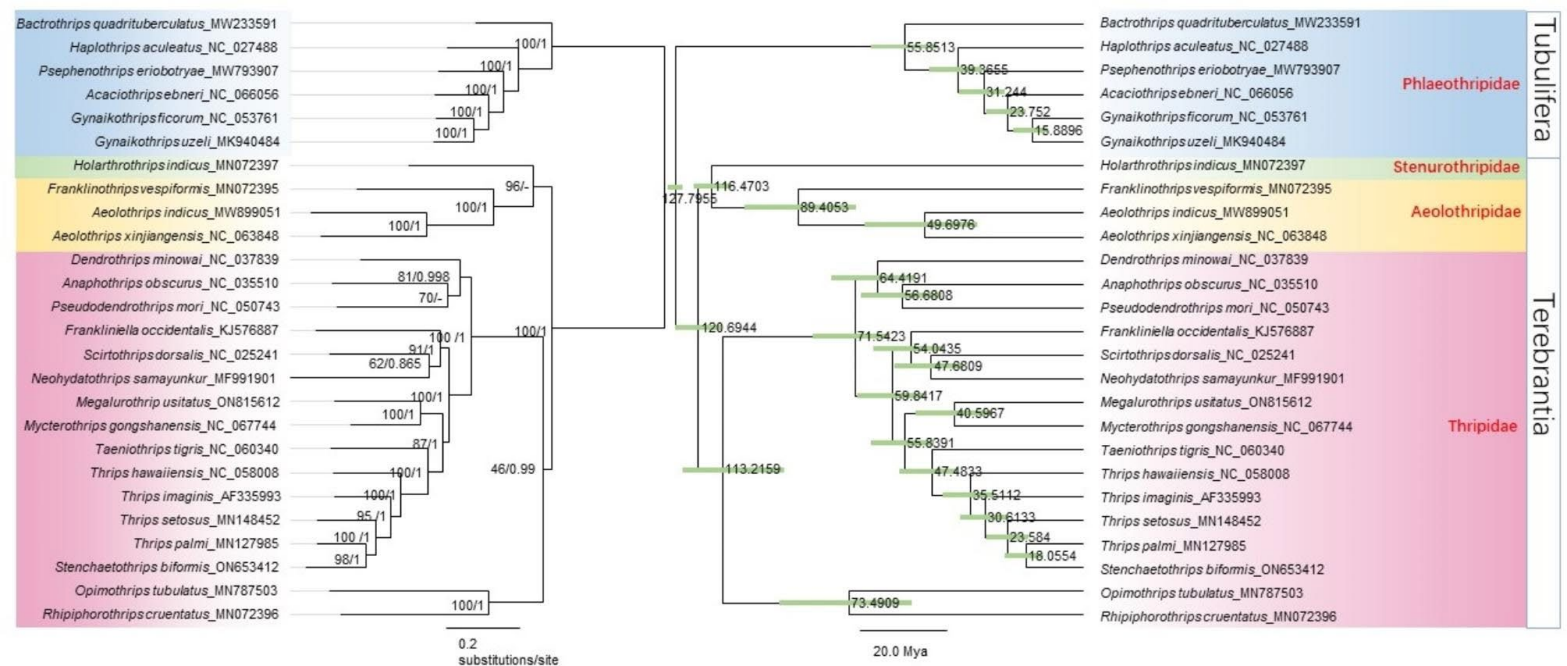
Left: Phylogenetic relationships of Thysanoptera. Maximum likelihood and Bayesian inference tree estimated from the concatenated data set of mitochondrial data (13 PCGs). Nodal support values are maximum likelihood bootstraps and Bayesian posterior probabilities. The species name is followed by NCBI accession numbers. Right: Divergence time estimation. The number next to each node represents the mean age (Mya). The bars on the nodes show the 95% HPD of the divergence times

The best partitioning strategy and models for ML and BI trees were in “Supplementary material” section. The phylogenetic analyses of the concatenated 13 protein-coding genes data set using BI and ML produced phylogenetic trees with similar topologies, and the differences occur mainly in nodes identified as weakly supported or collapsed. In this study, the ML tree are mainly used, and the BI tree are matched to the ML tree. Nodal support values of ML bootstraps and Bayesian posterior probabilitiesare shown in Fig. 1.

Phylogenetic results showed that monophyly was supported in both suborders Tubulifera and Terebrantia. In the Terebrantia, family Stenurothripdae and family Aeolothripidae were clustered into a clade, and Stenurothripdae was at the base of this clade. At this node, ML bootstrap support was high, but Bayesian posterior probabilities collapsed. Although family Thripidae exhibits a monophyly, the species *Opimothrips tubulatus* and *Rhipiphotothrips cruentatus* showed a paraphyly with other species in this family.

#### Divergence time deduction

The phylogenetic conclusion for this study was consistent between the gene trees and the time tree. (Fig. 1). The best model for the nucleotide substitution model is GTR + F + G4. The suborders Tubulifera and Terebrantia diverged in the Early Cretaceous period, approximately 127.80 Mya (95%HPD: 125.40-130.31 Mya). In this study, the divergence time of families Stenurothripidae and Aeolothripidae began about 116.47 Mya. The clade with the largest number of species in this study is Thripidae. Due to the large number of species, its main branches are incomplete, so the divergence time of this family should be earlier than 113.22Mya, and formed a large number of diverse species after 64.42 Mya, concentrated in the Early Tertiary. Species *Opimothrips tubulatus* and *Rhipiphotothrips cruentatus* of Thripidae became independent from the rest of this family and began to differentiate around 73.49 Mya. The ML and BI trees are nearly identical, with the exception of *Holarthrothrips indicus*, a species belonging to the family Stenurothripdae. This discrepancy may be attributed to differences in the conformation of phylogenetic trees or a lack of genetic information loci. Nevertheless, the phylogenetic relationship between the ML tree and time tree remains totaly consistent.

## Discussion

Insects originated in the early Ordovician period, about 479 Mya, and acquired the ability to fly at 406 Mya in the Devonian period [4]. During the Triassic period, insects began to diversify, with the emergence of the oldest ants, flies, moths and butterflies. Based on the fossil records of ancient extinct thrip species, the earliest thrips species with wings of the family Triassothripidae, appeared in the late Triassic [1, 2]. The fossil records of thrips from the Jurassic includes species from the families Liassothripidae and Karataothripidae [3]. A range of species of the family Hemithtipidae have been recorded from the Eocene [23, 24]. True thrips have been abundant since the early Cretaceous [2]. During this period, the sea floor expanded, the ancient land disintegrated, the mountain range formed, and the natural environment on earth developed towards diversity. Angiosperm bloomed, reptiles declined, mammals, birds, and bony fish rose. It was a time of significant change on Earth, including the extinction of dinosaurs and the rise of mammals.

Thus, the modern species of the families Phlaeothripidae, Aeolothripidae, and Thripidae in this study diverged and diversified in close proximity in time (about 65 − 23 Mya). The early Tertiary period lasted from about 65 million years ago to 23 million years ago. The current study provides a temporal reference for the origin and divergence of thrips. With the development of paleontology, we can combine the fossil calibration time to further estimate the accurate divergence time of the species of Thysanoptera.

## Conclusions

Fossils do not represent the only evidence of the past. There is a second record of the evolution of life, preserved in the genomes of all living organism. Combining time points and genomic information, using the “molecular clock” method, the divergence time of species can be inferred to form the time tree. The study used 13 mitochondrial coding protein genes to estimate the phylogenetic tree with divergence time of 26 species of the order Thysanoptera. The result shows that, most extant thrips diverged in the early Tertiary period, while most extinct thrips also appeared in this period. Therefore, it can be inferred that the early Tertiary is a period of renewal, differentiation and prosperity of thrips insects. The phylogenetic relationships in this study agreed with the morphological classification of the order Thysanoptera. The monophyly of the suborders Tubulifera and Terebrantia was clear, and the species of each family can be clustered separately, and the relationship between the species is well defined. So it was feasible to use multiple mitochondrial genes to increase genetic information loci and establish robust phylogenetic relationships between species.

Further studies could use more genetic data from living thrips species to refine the estimates of divergence times and improve our understanding of the evolution of this insect. The time tree reconstructed using two fossil calibrations shows that Phlaeothripidae and other families diverged around 127.80 Mya, but the Phlaeothripidae species in this study all diverged after 55.85 Mya. Stenurothripidae and Aeolothripidae diverged from Thripidae at about 120.69 Mya, furthermore Thripidae species in this study formed 73.49 Mya later. Fossils of ancient thrips date back to 130 Mya, or even earlier, but this study used mitochondrial genetic data from living thrips species, and most of these species began to form between 65 and 23 Mya, so this is the approximate date of the formation of living thrips.

### Electronic supplementary material

Below is the link to the electronic supplementary material.

Supplementary Material 1

Supplementary Material 2

Supplementary Material 3

Supplementary Material 4

Supplementary Material 5

Supplementary Material 6

## Data Availability

The direct links of the datasets during the present study are available in the NCBI database. The links have formed a table and submitted to the Supplementary material section. 1, *Acaciothrips ebner*, NC_066056, https://www.ncbi.nlm.nih.gov/nuccore/NC_066056.1/. 2, *Aeolothrips indicus*, MW899051, https://www.ncbi.nlm.nih.gov/nuccore/MW899051. 3, *Aeolothrips xinjiangensis*, NC_063848, https://www.ncbi.nlm.nih.gov/nuccore/NC_063848. 4, *Anaphothrips obscurus*, NC_035510, https://www.ncbi.nlm.nih.gov/nuccore/NC_035510. 5, *Bactrothrips quadrituberculatus*, MW233591, https://www.ncbi.nlm.nih.gov/nuccore/MW233591. 6, *Dendrothrips minowai*, NC_037839, https://www.ncbi.nlm.nih.gov/nuccore/NC_037839. 7, *Frankliniella occidentalis*, KJ576887, https://www.ncbi.nlm.nih.gov/nuccore/KJ576887. 8, *Franklinothrips vespiformis*, MN072395, https://www.ncbi.nlm.nih.gov/nuccore/MN072395. 9, *Gynaikothrips ficorum*, NC_053761, https://www.ncbi.nlm.nih.gov/nuccore/NC_053761. 10, *Gynaikothrips uzeli*, MK940484, https://www.ncbi.nlm.nih.gov/nuccore/MK940484. 11, *Haplothrips aculeatus*, NC_027488, https://www.ncbi.nlm.nih.gov/nuccore/NC_027488. 12, *Holarthrothrips indicus*, MN072397, https://www.ncbi.nlm.nih.gov/nuccore/MN072397. 13, *Megalurothrips usitatus*, ON815612, https://www.ncbi.nlm.nih.gov/nuccore/ON815612. 14, *Mycterothrips gongshanensis*, NC_067744, https://www.ncbi.nlm.nih.gov/nuccore/NC_067744. 15, *Neohydatothrips samayunkur*, MF991901, https://www.ncbi.nlm.nih.gov/nuccore/MF991901. 16, *Opimothrips tubulatus*, MN787503, https://www.ncbi.nlm.nih.gov/nuccore/MN787503. 17, *Psephenothrips eriobotryae*, MW793907, https://www.ncbi.nlm.nih.gov/nuccore/MW793907. 18, *Pseudodendrothrips mori*, NC_050743, https://www.ncbi.nlm.nih.gov/nuccore/NC_050743. 19, *Rhipiphorothrips cruentatus*, MN072396, https://www.ncbi.nlm.nih.gov/nuccore/MN072396. 20, *Scirtothrips dorsalis*, NC_025241, https://www.ncbi.nlm.nih.gov/nuccore/NC_025241. 21, *Stenchaetothrips biformis*, ON653412, https://www.ncbi.nlm.nih.gov/nuccore/ON653412. 22, *Taeniothrips tigris*, NC_060340, https://www.ncbi.nlm.nih.gov/nuccore/NC_060340. 23, *Thrips hawaiiensis*, NC_058008, https://www.ncbi.nlm.nih.gov/nuccore/NC_058008. 24, *Thrips imaginis*, AF335993, https://www.ncbi.nlm.nih.gov/nuccore/AF335993. 25, *Thrips palmi*, MN127985, https://www.ncbi.nlm.nih.gov/nuccore/MN127985. 26, *Thrips setosus*, MN148452, https://www.ncbi.nlm.nih.gov/nuccore/MN148452.
