# Supplementary material for: Extant thrips diverged in the early tertiary period
Source: BMC Genom Data. 2023 Aug 16;24:46. doi: 10.1186/s12863-023-01146-1 (PMC10433686; doi:10.1186/s12863-023-01146-1)
Supplement: Supplementary file 3 — Supplementary Material 3 [file 12863_2023_1146_MOESM3_ESM.pdf]

1, The best partitioning strategy and models for ML and BI trees for phylogenetic analysis:

IQ TREE best partitioning strategy and model：
1)GTR+F+I+G4: *ATP6*,
2)TPM2+F+ASC+G4: *ATP8*,
3)TIM+F+I+G4: *CO1*,
4)GTR+F+I+G4: *CO2*, *CO3*, *Cytb*,
5) GTR+F+I+G4: *ND1*, *ND3*,
6) TVM+F+I+G4: *ND2*, *ND6*,
7) GTR+F+I+G4: *ND4L*, *ND4*, *ND5*.

MrBayes best partitioning strategy and model：
1) GTR+I+G: *ND1*, *ATP6*, *ND3*,
2) GTR+G: *ATP8*,
3) GTR+I+G: *CO1*,
4) GTR+I+G: *CO2*,
5) GTR+I+G: *CO3*,
6) GTR+I+G: *Cytb*,
7) GTR+I+G: *ND2*,
8) GTR+I+G: *ND4L*,
9) GTR+I+G: *ND5*, *ND4* ,
10) GTR+I+G: *ND6*.
