# Supplementary figures and images for: Extant thrips diverged in the early tertiary period
Source: BMC Genom Data. 2023 Aug 16;24:46. doi: 10.1186/s12863-023-01146-1 (PMC10433686; doi:10.1186/s12863-023-01146-1)

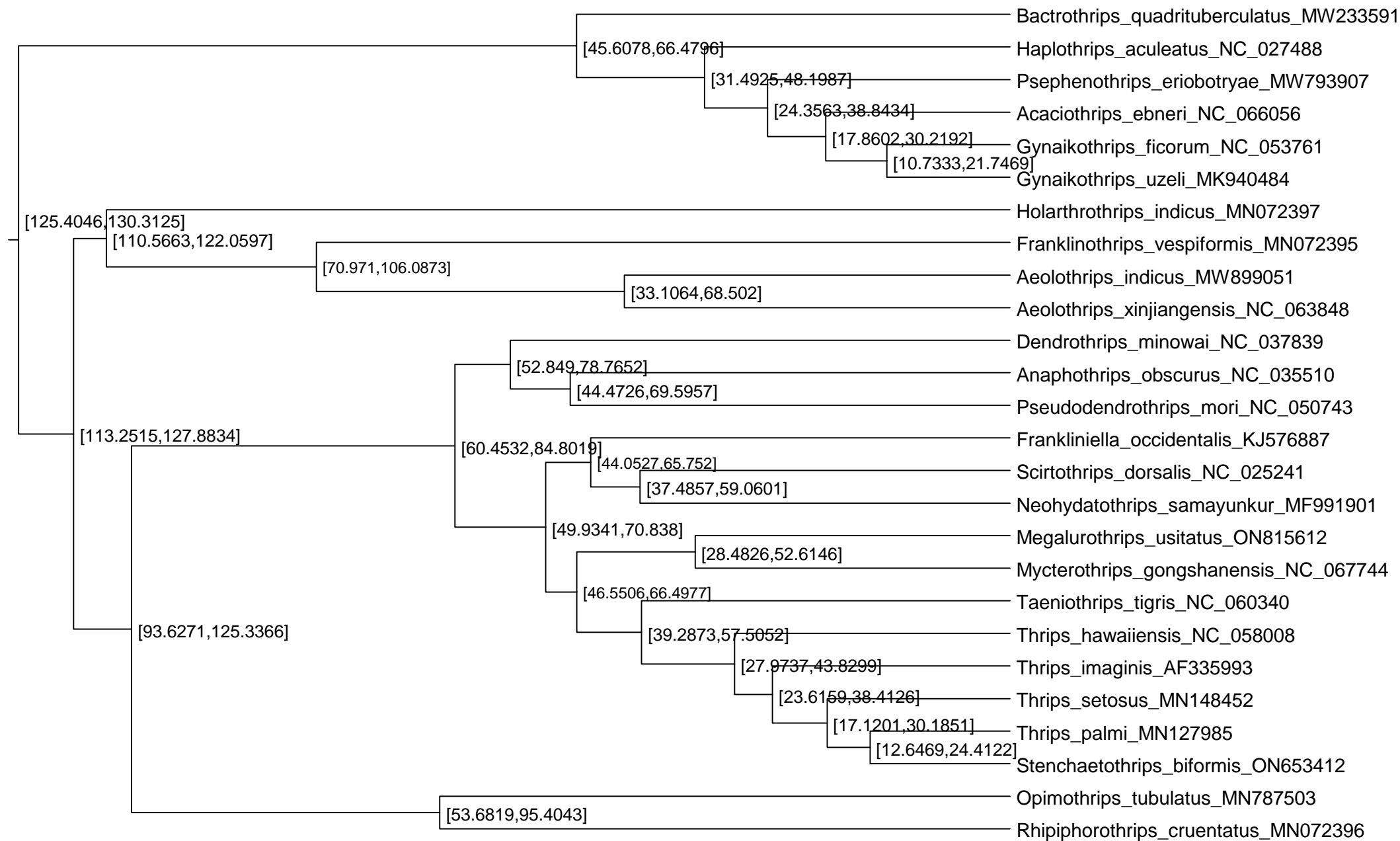

20.0 Mya

Supplement: Supplementary file 4 — Supplementary Material 4 [file 12863_2023_1146_MOESM4_ESM.pdf]

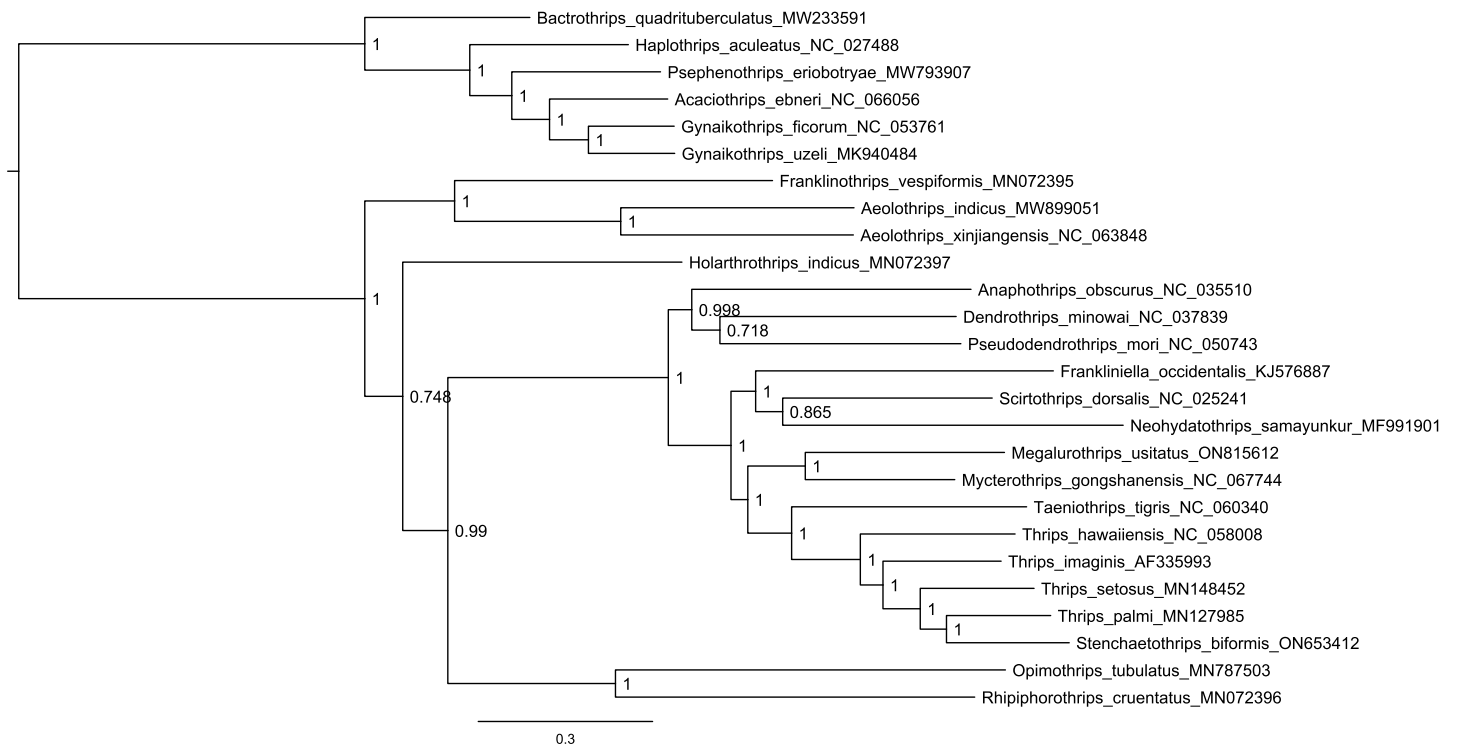

Supplement: Supplementary file 5 — Supplementary Material 5 [file 12863_2023_1146_MOESM5_ESM.pdf]
