# Supplementary material for: Extant thrips diverged in the early tertiary period
Source: BMC Genom Data. 2023 Aug 16;24:46. doi: 10.1186/s12863-023-01146-1 (PMC10433686; doi:10.1186/s12863-023-01146-1)
Supplement: Supplementary file 6 — Supplementary Material 6 [file 12863_2023_1146_MOESM6_ESM.docx]

Brief description about all the supplementary files

1, Supplementary Material 1 (The best partitioning strategy and models for ML and BI trees for phylogenetic analysis)

The Supplementary Material 1 is the best partitioning strategy and models for Maximum Likelihood (ML) and Bayesian Inference (BI) trees for phylogenetic analysis. Based on the data set, we used ModelFinder and PartitionFinder 2 to calculate the most suitable partitioning strategy and models for this phylogenetic analysis.

2, Supplementary Material 2 (figure, BI tree)

Supplementary Material 2 is the BI tree that preserves the branch length and posterior probabilities for reference.

3, Supplementary Material 3 (figure, Clock tree with 95% confidence interval)

Supplementary Material 3 is the clock tree with 95% confidence interval. The clock tree displayed alone is more obvious.

4, thrips species accession number and datasets direct links

These are the original links to mitochondrial genome data from 26 species of thrips publicly available at NCBI used in this study.

5, 26 thrips species-Mitochondrial gene extraction matrix (13 protein-coding genes in yellow)

A matrix of 13 protein-coding genes extracted from the mitochondrial genomes of 26 thrips species.
